# Does globalization in Turkey induce increased energy consumption: insights into its environmental pros and cons

**DOI:** 10.1007/s11356-020-08714-3

**Published:** 2020-05-01

**Authors:** Mfonobong Udom Etokakpan, Festus Fatai Adedoyin, Yorucu Vedat, Festus Victor Bekun

**Affiliations:** 1 Department of Economics, Eastern Mediterranean University, North Cyprus, via Mersin 10, Famagusta, Turkey; 2 Economics Department, Babcock University, Ikenne, Ogun State Nigeria; 3 Department of Accounting, Economics and Finance, Bournemouth University, Poole, UK; 4 Faculty of Economics Administrative and Social sciences, Istanbul Gelisim University, Istanbul, Turkey; 5 Department of Accounting, Analysis and Audit, School of Economics and Management, South Ural State University, 76, Lenin Aven, Chelyabinsk, Russia 454080

**Keywords:** Energy conservation, Pollutant emission, Globalization index, Turkey

## Abstract

Globalization is the paradigm shift to a more integrated world economy broadly shaping economies and societies around the globe. The wave of globalization is much more eminent on its impact on increased energy demand, knowledge and technology transfer, trade, and financial capital flows. The present study focuses on Turkey, a fast-emerging economy that is no exception to the wave of globalization. This current study explores the dynamics between ecological footprints, energy consumption, and real income level for the case of Turkey in a carbon-income function while accounting for other covariate like globalization to avoid omitted variable bias. The study data spans from 1970 to 2017 on an annual frequency basis. The stationarity properties of the outlined variables were investigated. Subsequently, the equilibrium relationship between the variables is confirmed by the battery of recent robust estimation techniques. While to detect the causality of direction among the variables, the Modified Wald test causality test is utilized. This study reveals that an increase in energy consumption in Turkey reduces environmental pollution by a magnitude of 0.37% in the short run and 0.43% long run, while an increase in economic expansion dampens the quality of the environment 0.42% and 0.72% on both short and long-run basis. This is indicative given that Turkey is more energy conscious and energy efficient, while a positive statistically significant relationship is observed between real income level and ecological footprint and globalization index. The causality analysis also supports the growth-induced energy consumption hypothesis. The study further offers policy direction for the energy sector in Turkey in the face of global interconnectedness.

## Introduction

Globalization in recent times has been up for discussion in many energy-environment pieces of literature due to the role and contribution to virtually all facet of endeavor. It is known to encourage technical innovation, improve living and environmental standards, boost total productivity through an increase in economic activity, and improve the environmental conditions. Globalization also allows the government to access to foreign efficient technologies to either import or export through international trade policies (Shahbaz et al. 2013 and 2017). While some scholars argue that globalization could be harmful to the environment and the economy through the transfer of pollution basically by exchanging the nonrenewable energy sources in a case where the other partner has weak environmental regulations, others posit that it can be beneficial where adequate regulations are ensured. This suggests strongly the reason globalization is either negatively related to growth and positively related to the environment (where carbon dioxide emissions are used as a proxy) or positively related growth and negatively associated with the environment (Snyder 2008). Globalization could enhance environment quality where a country reaches higher standards of living as a result of interaction with other countries (globalization), people’s consciousness increases, and accordingly they demand improved environmental quality.

The discussion between globalization and environmental degradation is on-going and contentious. The dynamics around globalization and the environment is not linear rather can take a different dimension. The globalization and environment dynamics can be categorized into three frequencies, namely, (a) scale effect, which asserts that globalization encourages economic growth and by extension energy consumption, which, in turn, increases environmental emissions (Cole 2006; Dedeoğlu and Kaya 2013); (b) composition effect suggests that globalization increases economic growth mainly due to shares of goods in the production processes using carbon-intensive techniques that are reduced; this, in turn, decreases the consumption of energy (Stern 2007). Lastly, the technique effect occurs when globalization decreases energy consumption and environmental degradation mainly due to the use of sophisticated technologies, technical know-how, and research and development (R&D) to boost the economic growth of a country (Antweiler et al. 2001; Dollar and Kraay 2004; Jena and Grote 2008).

The relationship between energy consumption, GDP, globalization, and ecological footprint is intertwined in such a way that it has a real impact on economic development. However, growing global concerns from international organizations as well as the call for the use of sustainable environmental policies by local and national governments have pushed to the fore a conservationist approach to the environment. However, the goal of maintaining sustainable environmental policies also competes with the level of energy consumption needed for large-scale industrial activities, especially in value-chain countries such as Turkey. This is because many countries are faced with the dilemma of attending to urgent energy consumption needs required to boost production while considering environmental sustainability without risking lagging in development projection. As a result, the integration of international markets through different avenues such as trade agreements has further accentuated the level of energy consumption as countries leverage international trade to access foreign markets.

The foregoing, therefore, raises the question of whether globalization has any inducement to energy consumption and an increase in economic output, as well as an impact on environmental degradation vis-à-vis the implication of achieving sustainable goals. Increasing research on the interconnectedness of energy consumption, GDP, globalization, and ecological footprint reveals that there is a possible case to be made that these variables interrelate in a way that they adversely impact the environment. Nonetheless, there is yet a need for more empirical evidence in this direction.

**Fig. 1:**
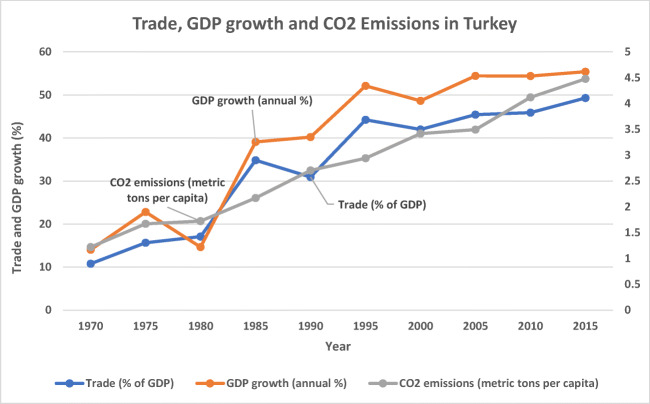
Trade (% of GDP), GDP growth (annual %), and CO_2_ emissions (metric tons per capita) in Turkey. Source: Authors compilation (Data: World Bank Development Indicator 2019)

**Fig. 2:**
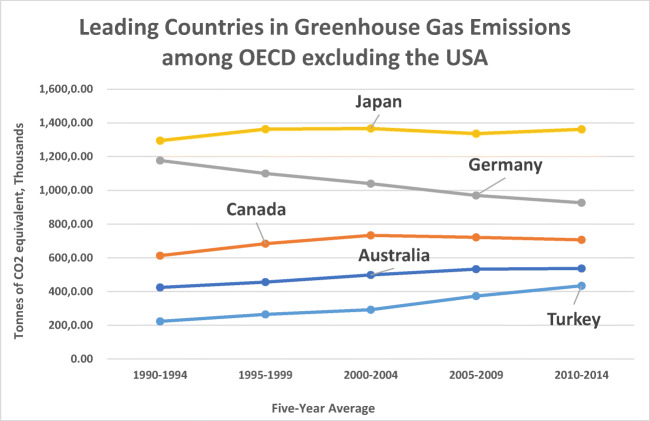
Greenhouse gas emissions (tonnes of CO_2_ equivalent, thousands) in OECD countries. Source: Author’s compilation (Data source: OECD Statistics 2019)

As an emerging economy, although characterized by some level of instability (Akadiri et al. 2019b), Turkey has continually sought economic development through increased external trade, diversification, and more importantly globalization. This positions Turkey as a significant premise for assessing how globalization affects energy consumption and how this influences the environment. More so, carbon emissions are the most influential factor in environmental degradation (Bekun et al. 2019), and globally, the risk of carbon emissions has drawn concerns from different angles. In fact, within the last 130 years, there has been a steady uptick of carbon dioxide emissions by 45% (Harvey 2018). For example, in the case of Turkey, CO_2_ emissions measured by metric tons per capita has been on the increase in the past five decades (Fig. 1), and emissions in Turkey represents the sixth largest among OECD countries (Fig. 2). As a result, the high level of carbon in greenhouse gases—making up 81% of greenhouse gases—has caused many governments to make a consensus to control the level of carbon (Owusu and Asumadu-Sarkodie 2016), which led to the Paris Agreement of 2015. Although Turkey, under the Paris Agreement, has undertaken to cut down carbon emissions by 21% before 2030, its trend of emissions shows some inconsistencies (Fig. 2), which further supports the classification of efforts by Turkey towards reducing carbon emissions as “critically insufficient” (Climate Action Tracker 2018).

Using Turkey as a case study, there is evidence for the inconsistency of policies towards achieving a sustainable economy, and this has led to complications in commitment to reducing carbon emissions (Akadiri et al. 2018). In fact, over the years, several variables have moderated this relationship in the bid to promote economic activity. The need to assess the role of external trade cannot be overemphasized, although a recent shred of evidence has magnified the role of tourism in Turkey. Some of these studies have focused on the growth of investment in tourism and tourism-related activities as a factor that contributes to the increase in demand for energy, which leads to increased CO_2_ emissions (Alola and Alola 2018; Pata 2018). Since tourism is a trade-in service and contributes to foreign exchange earnings, Turkey’s tourism industry as projected for 2019 expects 48.6 million tourists, with the tourism sector expected to create 3 million jobs (World Travel and Tourism Council 2018), and from empirical findings, activities of both tourists and firms in the industry also poses environmental hazard to the environment. As Fig. 2 shows, trade as a percentage of GDP has been on an upward trend, which is a further consequence of globalization in Turkey, which impacts significantly on the quality of the environment (Akadiri et al. 2019b).

Carbon emissions are a primary reason for environmental degradation and according to the International Energy Agency, 2019, Turkey’s carbon emissions in 2018 grew by 1.7%. This shows a serious need to address the possible contributing factors. Though Turkey is not the major contributor to carbon emissions, its bid to maintain economic stability may adversely affect its carbon-cutting guarantees under the Paris Agreement. This makes it imperative to first establish the connection between environmental impact and other variables such as globalization, energy consumption, and real income. From the above consideration, this study is motivated to theorize the following: (i) that environmental sustainability (using ecological footprint as proxy) can be impacted significantly by globalization led growth, (ii) Turkey’s environmental sustainability can be affected by induced energy consumption, and (iii) the various factors under consideration have some degree of relationship and dynamics among them. The use of ecological footprint accounting as a dependent variable in this study as opposed to the regular carbon dioxide (CO_2_) or greenhouse gas emissions is significant in contributing to the existing literature. The ecological footprint (EFP) covers a wider perspective of environmental degradation, which has been disregarded in the literature of energy and environment. This is a value added in expanding the frontier of knowledge concerning the literature, hence bridging the gap identified in the literature and further allowing for a robust discussion in the literature of energy and environment. The ecological footprint is unique in that it consists of certain qualities that are capable to account for natural essentials as well as economic development (Bello et al. 2018). The natural component of the EFP accounts for the following: forest reserves, fresh air, and availability of water resource and freshwater with the availability of arable farmland, which has the capacity and ability to support life and further ensures the terrestrial acidity and ecotoxicity of the ecosystem. This uniqueness distinguishes EFP from other proxies such as carbon dioxide emissions (CO_2_) and greenhouse gases (GHG) as it is employed in this study. The pollutant-environment and economic growth literature record that the use of EFP is rare, and as such, to the best of the author’s knowledge, this study serve as the link to bridge the gap as well improving the quality of discussion in the relevant literature (Katircioglu et al. 2018).

The rest of this paper will take the following sequence: “Literature review” will review related literature with a focus on the nexus between globalization, energy consumption, economic growth, and ecological footprint. “Methodological framework” will address the data sources and the methodology framework of the study; “Preliminary analysis” provides a preliminary analysis of the study. “Empirical results and discussion” presents empirical results and discusses this empirical result, while “Conclusion” consists of the conclusion and policy implication of the study based on the findings from the study.

## Literature review

### Globalization, energy consumption, and the environment

Globalization has been identified as one of the important factors that drive economic growth. Whether this directly impacts on the level of energy consumption of each country is yet to be found out as most prior research focused on economic growth solely. This economic growth was usually measured by GDP, GNP, GNI, employment, and real income. The pioneering work of Kraft and Kraft (1978) influenced the flurry of literature on defining the causality that influences economic growth and whether energy consumption plays an important role. The study of energy consumption, globalization, and the environment are relevant in this discourse as it provides a basis to understand if certain policies such as conservatism will adversely affect the economic growth of a country.

Several studies have also shown that development in urbanization and economic growth contributes to the pressure on energy consumption. Likewise, the fast growth of Turkey’s economy has been found to have a causality influence from the carbon emissions in CO2 (Lise 2006). Carbon emissions have an adverse effect on the environment due to its impact on environmental quality. Globalization has also been considered as an influence on energy consumption and environmental quality by Akadiri et al. (2019a, b, 2018). Therefore, as the world continues to increase its mobility in the bid to attain excellent growth performances, it becomes imperative to study the effect of energy consumption and economic growth on the environment.

Globalization is the process of global integration, which happens as a result of the exchange of worldviews, products, ideas, and other areas of culture in which national economies instill themselves into the global market, resulting in the pursuit of a common global economic goal. The influence of globalization has been shown from different perspectives to have a significant effect on economic growth and other development indicators such as real income. Globalization has also been examined to have affected economic growth, energy consumption, and the environment (Feridun et al. 2006).

The rising concern on the level of global warming coupled with unfortunate natural disasters has increased the level of consciousness around how we consume energy and whether energy consumption controls may result in lower real income or lower economic growth. This has also encouraged more studies into detecting the possible connection between energy consumption and economic growth.

There have been previous studies that assess the connection between the degradation of the environment, consumption of energy, and the growth of the economy (Destek et al. 2018; Ozcan et al. 2020, 2019; Tzeremes 2018). Adedoyin et al. (2020a) and Cetin et al. (2018) was able to establish the existence of a long-term connection between real per income capita and carbon emissions. The study established a unidirectional Granger causality from real per income capita to carbon emissions. Another study by Bojanic and Warnick (2019) assessed the impact of tourism on greenhouse gases (GHG) emissions in Turkey. Interestingly, they found that countries with higher levels of tourism suffer less GHG emissions in contrast to countries that have lesser or no component of tourism as their GDP. Balli et al. (2019) also examined the relationship between tourism and CO_2_ emissions, and their findings provide that tourism raises the level of CO_2_ emissions. Through the qualitative study of Song et al. (2018), research has established that there is a connection between tourism and economic globalization. Another study by Javid and Katircioglu (2017) found that social economic and political globalization played a major role in the influence of tourism development. These studies have thus established the connection between globalization and tourism. It, therefore, validates the purpose of this research, which is to detect the possible nexus between energy consumption, globalization, the environment, and economic growth. Brahmasrene and Lee (2017) were able to examine the effects of globalization, tourism, and industrialization on the environment. The study that focused on the case study of Southeast Asia between 1988 and 2011 established that there is a long-term relation between the examined variables and that tourism, globalization, and industrialization have a negative effect on the environment.

Although Akadiri et al. (2018) and Akadiri et al. (2019a) have previously assessed the relationship between globalization and carbon emissions, their study was not environment-centric. This research hypothesizes that the effect of globalization, though increases economic growth for a country like Turkey, harms the environment. The study of Turkey by Pata (2018) was able to conclude that there is a positive connection between per capita GDP, urbanization, and the reduction in per capita CO_2_ emissions in the long term. Globalization can also be seen as a factor from the perspective of foreign direct investment. Sarkodie and Strezov (2019) analyzed the interrelation between FDI, economic development, energy consumption, and the increase of GHG. Their study that focused on developing countries established that FDI increases economic development and CO_2_ emissions. However, Sarkodie and Strezov (2019) limited their source of data to only FDI inflows. This study intends to consider globalization in the extended form, that is, the study of Turkey will not be limited to Turkey’s FDI inflow only.

### Energy consumption and economic growth

The study of energy consumption has been found to influence economic growth (Udi et al. 2020).or may not in other instances, depending on the form of energy consumed (Kirikkaleli 2020). Energy generally comes in various forms such as fossil fuel and electricity. Ghosh (2002) implies that if electricity-led growth is supported by empirical support, the case can be made that conservation policies would be disastrous for the growth of the economy. Narayan et al. (2007) opined that if economic growth causes electric consumption, then there will be no adverse effect if electricity-related conservation policies are implemented.

Iyke (2015) admits that the uncertainty that surrounds the causality debate also covers electricity/energy consumption and economic growth. That is, there exists empirical evidence that shows a unidirectional causality from energy consumption to economic growth. The findings of Kumar Narayan and Singh (2007), Tsani (2010), and Bowden and Payne (2009) showed that energy consumption increases growth. Another argument shows empirical evidence that supports the view that economic growth influences energy consumption. This study is known as the conservation hypothesis, and studies by Ghosh (2002), Adom (2011), and Mozumder and Marathe (2007) find that energy conservation policies will not affect economic growth.

The bidirectional causality argument also argues that there is a two-way influence between energy consumption and economic growth (Adedoyin et al. 2020b). This argument posits that the two variables of energy consumption and economic growth could induce activity or growth either way. Masih and Masih (1997) made findings that support the bidirectional causality argument. The fourth argument supported by studies such as Cheng (1995) argues that there is no causal relation between the two concepts of energy consumption and economic growth.

The lack of certainty as posited by Iyke (2015) may be attributable to different reasons, one of which is the nature of variables adopted in the research. Ebohon (1996) recognized this in his study underlining that factors such as supply constraints and price rigidity in developing countries render some studies meaningless. In this study, we will improve on some of these initial case studies by focusing on globalization, real income, energy consumption, and ecological footprint.

Sekantsi and Timuno (2017) assessed the impact of financial development on energy consumption in Botswana, using the Autoregressive Distributive Lag (ADRL) and Error Correction Model (ECM). They concluded that economic growth, financial development, and industrialization increase the consumption of energy and electricity in Botswana. Akinlo (2008) conducted a broad study of 11 SSA countries, using the ADRL test and concluded that energy consumption has a positive effect on economic growth. This study was, however, unable to draw a similar conclusion in its findings. Some findings showed the existence of the neutrality hypothesis, while others showed a bidirectional relationship. Odhiambo (2010) made distinct findings on the unidirectional causality between economic growth and energy consumption, from a panel of three SSA countries. Menyah and Wolde-Rufael (2010) analyzed the South African market adopting 5 variables: economic growth, pollutant emissions, labor, capital, and energy consumption. Their study concluded that there is a long-run and short-run connection between the variables with a significant impact found between pollutant emission and economic growth.

The study of the impact of economic development is important to determine how factors of development can be measured with their cumulative impact whether in the short term or the long term. The increasing competition for land, labor, and capital as factors for production has also extended to a new variable, energy. The consumption of energy has been shown through various studies to be pivotal to economic development. However, new challenges are being raised to the way countries sustainably deploy energy consumption in a way. This research attempts to improve the discourse on the relationship between economic growth and energy consumption. To improve on previous literature, this study adds the variables of ecological footprint and real income to detect whether energy conservation policies may have a negative impact on economic growth in Turkey.

This study analyzes the causality effect between the identified variables during the course of 1970–2017 for Turkey with a focus on the impact of energy consumption, ecological footprint, and real income on the environment, which is underscored by the rising need to examine the impact of economic activities on the climate. Advanced interconnectedness among countries has empowered countries’ economies and promoted inflow and outflow of trade. Increasing ease of access in trade has also been closely associated with more energy consumption. The level of energy consumption has also led to more contractions in the management of environmental sustainability. Pao and Tsai (2010) studied the BRIC countries except for Russia. Their findings also established a long-run connection between carbon emissions, energy usage, and real output from the BRIC countries. Besides, Pao and Tsai (2010) recommend that the balance between the environment and energy consumption in developing countries be maintained through the creation of energy-efficient policies and systems. While Owusu and Asumadu-Sarkodie (2016) examined the impact of labor, capital resources, and other production components and found that the contribution of human activities to the development of the world economy has been stimulated, other studies have focused solely on finding the causal link for between energy consumption and economic growth. Many have established that the determination of the causal link is not stable. Balcilar et al. (2010) opine that the objective for testing the connection between energy consumption and real GDP is based on the need for energy conservation policies. They further opine that if a unidirectional causality link is found to flow from energy consumption to development, then energy conservation policies will harm the growth (GDP) of the economy. For example, according to Ozturk and Acaravci (2010), using the Granger causality test, the existence of a unidirectional causality shows a movement from economic growth to energy consumption, which can also be extended to other macroeconomic variables such as employment. The volume of studies on energy consumption-growth nexus shows that without a doubt, the nexus is an interesting and regular research discourse in the literature.

Based on the highlighted literature, in summary, as many scholars have admitted, the uncertainty of a nexus between the concept of economic growth and energy conservation remains since the seminal study of Kraft and Kraft (1978) a significant challenge; hence, the current study seeks to extend research in the area of an holistic investigation with recent data for the case of Turkey with an interesting energy mix with more emphasis on the role of globalization effects.^1^ Following the reviewed pieces of literature, the contributions of this study to body of knowledge includes: (a) Firstly, this study incorporates globalization index, which considers economic, social, and political aspects of globalization, energy consumption, real income, and ecological footprint, which also considers cropland, grazing, forest, fishing, CO_2_ emissions, and infrastructure footprint, to investigate whether globalization induces energy consumption and the effect on the environmental quality in Turkey throughout 1970–2017. The choice of the variables was informed by the United Nations Sustainable Development Goals (SDGs) agenda of access to clean affordable energy and mitigation of climate change issues respectively targeted to be achieved by 2030. (b) Secondly, this study has incorporated that the ecological footprint with its unique characteristics as a dependent variable in its data estimation technique is rare in similar studies for Turkey. Most studies use carbon dioxide emissions (which is one of the components of EFP) as the proxy for environmental quality. (c) Finally, studies of this sort are timely and worthwhile for policymakers and energy practitioners for ample policy design given global crusade for cleaner energy sources exploration (Table 1).

**Table 1`:** Summary of literature on energy consumption and economic growth

S/N	Author	Period	Region	Methodology	Variables	Direction of causality
1	Dlamini et al. (2015)	1971–2009	South Africa	Bootstrap rolling- window	ELC and GDP	ELC → GDP for two sub-periods
2	Ebohon (1996)	1960–1984	Tanzania, Nigeria	Granger Causality	EC and GDP	EC ↔ GDP
3	Shahbaz et al. (2015)	1980–2012	12 African Countries	FMOLS, Pedroni cointegration test and VECM	EI, CO_2_ and Real GDP	GDP↔ CO_2_, EI → CO_2_
4	Morimoto and Hope (2004)	1960–1998	Sri Lanka	Granger causality	EP and Real GDP	EP → GDP
5	Balcilar et al. (2010)	1960–2006	G-7 Countries	Bootstrap Granger non-causality test	EC and Real GDP	EC → GDP for only Canada, there is no causal links between energy consumption and economic growth for the other countries
6	Nazlioglu et al. (2014)	1967–2007	Turkey	ARDL approach, linear and nonlinear Granger causality test	ELC and GDP	ELC ↔ GDP for linear causality test, no non-linear causality between ELC and GDP
7	Al-Mulali et al. (2016)	1980–2012	Kenya	ARDL bounds testing	GDP, trade openness, urbanization	N/A
8	Osabuohien et al. (2014)	1995–2010	Africa	DOLS, Pedroni cointegration.	EC per capita, GDP, GDP square	N/A
9	Shahbaz et al. (2015)	1980–2012	African countries	FMOLS, Pedroni, VECM	GDP, CO2, energy intensity	Y ↔ C E → C
10	Shahbaz et al. (2013)	1965–2008	South Africa	ARDL bounds testing, VECM	GDP, CO2, EC	Y ↔ C E → C,
11	Iyke (2015)	1971–2011	Nigeria	VECM, Granger causality	GDP, ELC	E → Y
12	Kwakwa (2017)	1971–2012	Egypt	Engle-Granger, FMOLS	GDP, ELC	E → Y
13	Tamba et al. (2017)	1971–2013	Cameroon	Johansen cointegration, VAR, Granger causality	GDP, ELC	N/A
14	Sekantsi and Timuno (2017)	1981–2011	Botswana	ARDL, VECM	GDP, EC, FD, IND	E → Y
15	Sekantsi and Okot (2016)	1973–2012	Lesotho	Cointegration, VECM	GDP, EC, FD, IND	EC ↔ GDP
16	Sarkodie (2017)	1980–2030	Ghana	ARIMA forecasting	GDP, ELC	EC grows from 8.52 Kwh billion to 9.56 billion Kwh 2030
17	Akinlo (2008)	1980–2003	11 SSA African countries	ARDL, VECM, Granger causality	EC and Real GDP	Mixed findings from countries with diverse policy implications
18	Ghosh (2002)	1950–1997	India	Engle-Granger causality test	ELC and GDP	GDP → ELC
19	Mozumder and Marathe (2007)	1971–1999	Bangladesh	Johansen cointegration test and Granger causality test based on VECM	ELC and GDP per capita	GDP → ELC
20	Lee and Chang (2005)	1954–2003	Taiwan	Gregory and Hansen structural break test and Granger causality test	EC (coal, gas, oil, and electricity) and Real GDP	EC → GDP
21	Kraft and Kraft (1978)	1947–1974	USA	Granger causality approach	GNP, EC	Y → EC
22	Solarin and Shahbaz. (2013)	1971–2009	Angola	ARDL, VECM	Capital, Labor, ELC	ELE → Y
23	Belloumi (2009)	1971–2004	Tunisia	VECM, Granger causality	ELC and GDP	EC → Y
24	Amusa and Leshora (2013)	1981–2010	Botswana	ARDL bounds testing	ELC and GDP	ELE → Y
25	Odhiambo (2009)	1971–2006	Tanzania	ARDL bounds testing	ELC and GDP	ELE ↔ GDP
26	Jumbe (2004)	1970–1999	Malawi	VECM, Granger causality approach	ELC and GDP	ELE → Y

“→, ↔” indicate unidirectional and bidirectional causality, respectively

*ELC* electricity consumption, *EC* energy consumption, *FD* financial development, *GDP* gross domestic product, *CO*_*2*_ carbon dioxide emissions, *FDI* foreign direct investment, *EI* energy intensity, *EP* energy production, *E* employment, *NA* no causality in either direction

## Methodological framework

This section of the study will focus on data sources, units of measurement, and the procedures applied in the estimation of the variables selected for the study.

### Data

This study uses time series framework analysis in investigating and determining the role of globalization-led growth in enhancing an increase in energy consumption, bearing in mind the environmental implications and consequences. The variables of interest used in the analysis include energy consumption (EU), real income (RDGP), globalization index (GLO), and ecological footprint (EFP), which is a measure for environmental quality for the case of Turkey—a fast-emerging economy in the Middle East where there is a high impact of industrial activities. Data were sourced from the World Bank Development Indicator, 2019, Global Footprint Network National Footprint Account (2018 edition), and KOF Swiss Economic Institute Database (KOF 2017) and were restricted between periods of 1970–2017 basically as a result of data availability.

Furthermore, the current study utilizes real income (RGDP) as proxy for economic growth measured at constant 2010 USD, whereas globalization index is proxied to account for economic, social, and political dimensions of globalization as developed by Dreher (2006), while energy consumption is composed of energy production and consumed before prior transformation in oil equivalent kilogram terms. This study will follow three empirical procedures in its analysis. First, examine and determine the order of integration and as well as the asymptotic stability of the variables using the stationarity tests. The basic reason for this is to avoid spurious regression. Second, with the use of the ARDL bounds cointegration test, a long-run equilibrium relationship of the data series is established. Lastly, the direction of causality was examined and determined with the use of the Toda-Yamamoto causality test via Block exogeneity procedure. Table 2 gives a summary of variables, description, unit, and source.

**Table 2:** Data description, source, and unit of measurement

Variable	Unit of measurement	Source
Ecological footprint (EFP)	The global hectare of land	GFP
Energy consumption (EU)	Oil equivalent per in kg	WDI
Globalization index (GLO)	Percentage	KOF
Real gross domestic product (RGDP)	Constant 2010n$ USD	WDI

Author’s compilation

### Model specification

This paper is built on the foundation of the study of Shahbaz et al. (2016) and such the functional expression is presented as:

1 $$ \mathrm{EFP}=\mathrm{f}\ \left(\mathrm{EU},\mathrm{GLO},\mathrm{RGDP}\right) $$

The transformation of the above equation into the logarithmic series is necessary to ascertain homoscedasticity in the above expression.

2 $$ \mathrm{LnEF}{\mathrm{P}}_t=\beta +{\beta}_1\mathrm{LnE}{\mathrm{U}}_t+{\beta}_2\mathrm{LnGL}{\mathrm{O}}_{\mathrm{t}}+{\beta}_3\mathrm{LnRGD}{\mathrm{P}}_{\mathrm{t}}+{\varepsilon}_t $$

From the above expression, *β* denotes the constant variable, whereas the partial slope parameters are represented by *β*_1_, *β*_2_, and *β*_3_. From the above model, a priori expectation conforms to theory and empirics. The expectation that *β*_1_, *β*_2_, and *β*_3_ individually are greater than zero holds. This implies that energy consumption in Turkey positively contributes to the quality of the environment hence the *β*_1_ > 0 basically due to the use of modern and clean energy sources. Also, globalization plays a significant role in contributing to the high environmental quality as *β*_2_ > 0 represents. Lastly, positive sign is expected for *β*_3_ (i.e. *β*_3_ > 0). This means that economic growth is positively related to an environment free from degradation. This is usually the case for developed economies who are not just interested in improving output but also ensure that the environment is not traded-off for output.

### Testing for stationarity

Testing for stationarity is indispensable among variables in a series to establish the order of integration. The conventional unit root tests such as Elliott et al. (1996), Phillips and Perron (1988), and Augmented Dickey-Fuller test (Dickey and Fuller (1981) are known for turning out inconsistent and invalid estimates when confronted with a structural break in data series because of its deficiency in accounting for structural breaks. In a situation where an economic dataset is characterized by structural breaks as it is a norm with time series data, Zivot-Andrews with its unique characteristic of capturing structural break uniquely is usually complemented with the conventional unit root tests.

Zivot-Andrews test model can be expressed as345

where *DU*_*t*_ denotes the dummy variable showing the shift that occurs at a specific point of the potential breaks whether at the intercept, trend, or both. *Y*_*t-*1_ represents the first lag of the variables being tested. The null hypothesis of Zivot-Andrews unit root *H*0 : *β* > 0 is tested against the alternative of stationarity *H*1 : *β* < 0. This means that failing to reject the null hypothesis validates unit-roots presence, whereas the ability to reject the null hypothesis ascertains stationarity.

### Computing cointegration relationships

The processes of a testing cointegration relationship between variables are numerous as documented in the econometric literature. Long- and short-run are the basic ranges of the cointegration relationships (Carrion-i-Silvestre and Sansó 2006; Engle and Granger 1987; Gregory and Hansen 1996; Johansen 1991; Johansen and Juselius 1990; Phillips and Ouliaris 1990). However, there is need for a consideration of a more robust result since the earlier mentioned cointegration tests usually give different conclusions, hence the reason for individually exploring the test statistics of Bayer and Hanck (2013), Boswijk (1995), Banerjee et al. (1998), Engle and Granger (1987), and Johansen (1991).

#### Autoregressive distribution lag technique

Considering the following variables (ecological footprint, energy consumption, globalization index, and real gross domestic product) under investigation, there is a need to employ the autoregressive distribution lag (ARDL) bounds test, which is an efficient and robust technique in ascertaining cointegration in small samples. The unique feature of this approach is the dynamics of fitted regression at the same time with the error correction model associated with the long-run and short-run. This approach can determine the unknown order of integration of series whether it is I (0) or I (1). The error correction model (unrestricted version) assumes endogeneity of all variables, and it is specified as:

6 $$ \varDelta Y={\delta}_0+{\delta}_1t+{\beta}_1{y}_{t-1}+\sum \limits_{k=1}^Z{\gamma}_1{v}_{kt-1}+\sum \limits_{n=1}^X{\varphi}_n\varDelta {Y}_{t-n}+\sum \limits_{k=1}^Z\sum \limits_{n=1}^X{\mu}_{kn}\varDelta {V}_{kt-n}+\theta {D}_t+{\varepsilon}_t $$

The exogenous variable is denoted by *Dt*, which captures structural breaks in the framework, whereas *Vk* stands for the vector. The null hypothesis of no cointegration is usually validated by the *F* statistics computed from the bounds test. The following scenarios exist when making this decision: (i) if *F* value computed is greater than the upper bounds of the critical values reported, then the rule is to reject null hypothesis of no cointegration, (ii) should the *F* value lies within the lower and upper bounds, the decision is inconclusive result, and finally (iii) should the *F* value lies below the upper bounds, this is a case of no cointegration. The specification of the hypotheses for bounds test is expressed below:

$$ {H}_0:{\beta}_1={\beta}_2=\dots \dots ={\beta}_{k+2}=0 $$

$$ {H}_1:{\beta}_1\ne {\beta}_2\ne \dots \dots \ne {\beta}_{k+2}\ne 0 $$

where the *β*s denotes the *F* statistic values from the bounds test that is compared with the lower and upper bounds values to ascertain which hypothesis to reject.

#### Bayer and Hanck combined cointegration test

The field of statistics and econometrics have over the years documented the various techniques with regard to cointegration (equilibrium) analysis. Bayer and Hanck (2013) methodology is a recent development to cointegration accounted for the shortcomings of the previous test. The Bayer and Hanck (B-H) have a unique characteristic to combine single and multiple procedures from individual test statistics. This is responsible for the robust results from B-H to cointegration estimations. Boswiik and Banerjee test and Johansen, Engle, and Granger test provide the foundation upon which B-H is predicated. Below is the expression of the statistical computation of B-H:

7 $$ \mathrm{EG}-\mathrm{JOH}=-2\left[\ln \left({P}_{\mathrm{EG}}\right)+\ln \left({P}_{\mathrm{JOH}}\right)\right] $$

8 $$ \mathrm{EG}-\mathrm{JOH}-\mathrm{BO}-\mathrm{BDM}=-2\left[\ln \left({P}_{\mathrm{EG}}\right)+\ln \left({P}_{\mathrm{JOH}}\right)+\ln \left({P}_{\mathrm{BO}}\right)+\ln \left({P}_{\mathrm{BDM}}\right)\right] $$

where P_EG_, P_JOH_, P_BO_ *and P*_BDM_ denote the various corresponding individual cointegration probability test values. The B-H test is reported as a null hypothesis when there is no cointegration. In the case where the fisher test statistics is greater than the outlined critical values, then the null hypothesis is rejected and reported as cointegration among the variables of interest.

### Granger causality approach

It is a necessary condition to determine the direction of causality between variables, though it is a norm that a traditional regression does not necessarily imply causal relationships. Nevertheless, it is useful and needed by policymakers and stakeholders especially with regard to their predictability powers among the variables of interest. When *X* granger causes *Y*, it implies that in its sum the realizations (both present and past) of the *X* variable is a good predictor of variable *Y*. The bivariate form of the above can be expressed as follows:

9 $$ \kern3.5em {X}_t={\delta}_0+{\delta}_1{X}_{t-1}+{\delta}_2{Y}_{t-1}+{\varepsilon}_t $$

10 $$ {Y}_t={\delta}_0+{\delta}_1{Y}_{t-1}+{\delta}_2{X}_{t-1}+{\varepsilon}_t $$

From the above equations, the null hypotheses are usually tested against the alternative hypotheses (Eqs. 9 and 10). Granger causality can be reported in the following ways: (a) unidirectional, which means interaction of the variables from *X* to *Y* or otherwise; (b) bidirectional, implying feedback relationship among the variables of interest. This relationship can either flow from *X* to *Y* and vice versa; and (c) neutrality denotes no causal relationship or interaction between variables *X* and *Y*.

The current study adopts the use of the modified Wald stat (MWALD) advanced by Toda-Yamamoto (1995) for the detection of causality analysis for the study outlined variables. The Toda-Yamamoto (TY, hereafter) is pronounced for its merit over the conventional Granger causality test. The TY methodology is unique irrespective of the series order of integration. The TY test is built on the VAR framework with (*k + dmax*), where *dmax* represents the maximum order of integration and *k* is the optimal lag order.

## Preliminary analysis

**Fig. 3:**
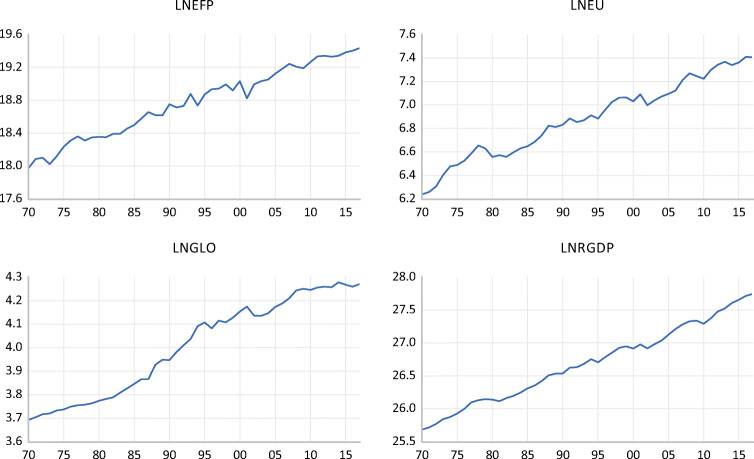
Graphical plot of variables under review

In time series econometrics modeling, it is always pertinent to explore the graphical display of the choice variables, basic summary statistics, and pairwise correlation analysis. This is critical for adequate modeling methodology. Figure 3 presents the pictorial display of the interest variables. All series exhibit a positive trend over the investigated period (1970–2017). This outcome is insightful. As the Turkish economy grows (RGDP), energy intensification (EU) in the current wave of globalization (GLO) grows. These positive trends deplete the quality of the environment as we see all variables grow at almost the same pace. This raises concerns for government administrators given Turkey is a fast-emerging economy. Subsequently, Table 3 presents the basic summary statistic of the variables with real GDP with the highest average and maximum value followed by ecological footprint, and the globalization index is the lowest average over the investigated period. All series show negative skewness except economic growth, while all series reveal a great departure from their means, which are reported by the standard deviation. The pairwise correlation between the variables is presented in Table 4, where we observe a strong statistical positive relationship between all variables under consideration. For instance, we observe a positive relationship between all dimensions of globalization and economic growth. This implies that as the world economies are all interconnected, it spurs economic progress and engenders intensification of energy consumption. The aforementioned assertion from the pairwise analysis is not sufficient to validate these prepositions given that the results of Pearson correlation are mere relationship base. Thus, the need for further econometric analysis is considered in other sections to either refute or validate these positions.

**Table 3:** Descriptive statistics

	LNEFP	LNENU	LNGLO	LNRGDP
Mean	18.76974	6.884355	4.005581	26.68953
Median	18.78766	6.884564	4.059123	26.69325
Maximum	19.43535	7.409355	4.278311	27.74659
Minimum	17.97528	6.237469	3.692567	25.68336
Std. Dev.	0.426561	0.327894	0.204415	0.588589
Skewness	− 0.145513	− 0.129699	− 0.154309	0.065250
Kurtosis	1.849143	2.037853	1.467100	1.933279

Author’s compilation

**Table 4:** Analysis of correlation matrix

	LNEFP	LNEU	LNGLO	LNRGDP
LNEFP	1.0000			
t-statistic	–			
Prob. Value	–			
LNEU	0.9847*	1.0000		
t-statistic	38.2714	–		
Prob. Value	0.0000	–		
LNGLO	0.9779*	0.9745*	1.0000	
t-statistic	31.7459	29.4338	–	
Prob. Value	0.0000	0.0000	–	
LNRGDP	0.9886*	0.9932*	0.9779*	1.0000
t-statistic	44.5963	57.7178	31.7348	–
Prob. Value	0.0000	0.0000	0.0000	–

Author’s compilation. Asterisk (*) denotes 1% significant rejection level

Furthermore, the present study proceeds to investigate the unit root properties of the underlined variables with the aid of conventional ADF and PP unit root test in conjunction with Zivot and Andrews unit root test in Table 6. Tables 5 presents the unit root test results where all variables are nonstationary at the level form but, after the first difference, all became stationary as reported by both the ADF and PP results. The ZA unit root test with a single structural break date validates the outcomes of ADF and PP that all outlined variables are the first difference stationary over the sample period for the case of Turkey (Table 6).

**Table 5:** Results of unit root test (without break)

Models	Variables	At level		At 1st difference		Decision
		t-statistic	Prob	t-statistic	Prob	
ADF test	LnEFPt	− 0.8020	0.8091	− 10.7935	0.0000*	I(1)
	LnEUt	− 1.2657	0.6377	− 6.4567	0.0000*	I(1)
	LnGLOt	− 0.8773	0.7868	− 6.3593	0.0000*	I(1)
	LnRGDPt	0.1052	0.9629	− 6.5592	0.0000*	I(1)
PP test	LnEFPt	− 1.2688	0.6363	− 14.9011	0.0000*	I(1)
	LnEUt	− 1.2998	0.6221	− 6.4477	0.0000*	I(1)
	LnGLOt	− 0.8774	0.7868	− 6.3509	0.0000*	I(1)
	LnRGDPt	0.1177	0.9638	− 6.5558	0.0000*	I(1)

Asterisk (*) denotes 1% significance level of rejection

**Table 6:** Unit root output (with single break date)

	Level			First difference			Decision
	ZA_I_	ZA_T_	ZA_B_	ZA_I_	ZA_T_	ZA_B_	
LNEFP	− 6.4114	− 5.8291	− 6.3521	− 7.2493	− 6.9104	− 7.2658	I (0)
Break date	2001	1994	2001	2003	2007	2003	
Lag	2	4	4	4	4	4	
LNEU	− 3.6293	− 3.3275	− 4.2326	− 6.3661*	− 6.2830*	− 6.5440*	I (1)
Break date	2000	2007	1979	1979	1981	1983	
Lag	4	4	4	1	1	1	
LNGLO	− 3.5726	− 2.4083	− 3.1999	− 6.9737*	− 7.0063*	− 7.4505*	I (1)
Break date	1988	2000	1988	1988	1993	1996	
Lag	4	4	4	4	4	4	
LNRGDP	− 4.0394	− 4.6397	− 5.3536	− 6.4135*	− 6.4000*	− 6.4696*	I (1)
Break date	1999	2010	2002	2005	2003	1999	
Lag	3	3	3	4	4	4	

LNGLO represents globalization index, LNEU is electricity consumption, LNEFP is ecological footprint and LNRGDP is the gross domestic product at constant prices. The variables are in their natural logarithms. ZA_B_ denotes the model with a break in both the trend and intercept, whereas ZA_T_ and ZA_I_ are for models with a break in trend and intercepts respectively. A 1% significance level is denoted with an asterisk

## Empirical results and discussion

The preliminary analysis hints on the relationship between the outlined variables under review. Before the investigation of the long-run (equilibrium) relationship between the outlined variable. The need to explore the optimum order of lag length is pertinent to avoid spurious analysis. Table 7 renders the parsimonious lag order choice as Schwartz Bayesian Information criterion (SIC) as optimum for the rest of the study. The econometrics literature documents series of cointegration model especially in the 1980s. More recently, one of the latest and novel is that the Bayer and Hanck (2013) is conducted for cointegration analysis. Table 8 reports that the Bayer and Hanck combined cointegration results with overall results indicating a cointegration relationship among the variables over the sampled period at (*p* < 0.05) statistical level. For robustness check, the ARDL bounds test to cointegration is conducted at the bottom of Table 8, and the ARDL bounds test results with *F* statistics (*p* < 0.01) statistical threshold align with the B-H combined cointegration results. This implied that there is a converge and equilibrium relationship between the variables under review.

**Table 7:** Parsimonious lag order

Lag	LogL	LR	FPE	AIC	SC	HQ
0	200.3323	NA	1.56E-09	− 8.924198	− 8.7619	− 8.8640
1	389.8531	335.9685	5.90E-13	− 16.8115	− 16.0005*	− 16.5107*
2	410.1697	32.3219*	4.94E-13*	− 17.0077*	− 15.5479	− 16.4663
3	423.4891	18.7682	5.86E-13	− 16.8858	− 14.7772	− 16.1039
4	438.6544	18.6119	6.71E-13	− 16.8479	− 14.0905	− 15.8253

*HQ* Hannan Quinn, *AIC* Akaike information criterion, *SC* Schwarz information criteria, *FPE* final prediction error, *LR* sequential modified LR statistic

**Table 8:** Bayer and Hanck result

Fitted model	EG-JOH	EG-JOH-BO-BDM	Cointegration decision
LnEFP = f (LnEU, LnGLO, LnRGDP)	20.405**	20.624**	Yes
Critical values (5%)	10.637	20.486	Yes

**Fig. 4:**
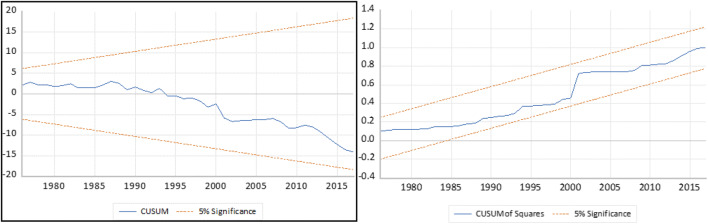
Stability test of CUSUM and CUSUMSQ

To further buttress the magnitude of the equilibrium relationship, the ARDL short- and long-run regression is fitted with an ecological footprint as the dependent variable. In Table 9, the speed of convergence or error correction term (ECT) is in harmony with earlier mentioned cointegration. However, in the case of disequilibrium, convergence pace is at approximately 58.29% on an annual basis with the contribution of model regressors, namely, globalization, economic growth, and energy consumption. From Table 9, we observe a positive relationship between all dimensions of globalization and quality of the environment in Turkey in the long run and negative relationship in the short run. This suggests that the wave of globalization should be checked with caution even though it is desirable in the short run but comes with its environmental implications like depletion of quality of the environment in the long run. This outcome is consistent with the study of see (Feridun et al. 2006; Akadiri et al. 2019a, b; Shujah-ur-Rahman et al. 2019). This revelation is a call for a more proactive step on the part of the Turkish government officials on her macroeconomic indicators in terms of interaction with the rest of the world to mitigate against the adverse effect of interactions with other economies. Furthermore, economic expansion exerts a positive and statistically positive impact on the environment in Turkey in the long run. This is indicative as Turkey is reputed as an emerging economy with much focus on macroeconomic stabilization and economic growth rather than an emphasis on pollutant emission. This implies that the Turkish economy is at her scale stage of its growth trajectory where the emphasis is on output relative to the quality of the environment (Balsalobre-Lorente et al. 2018; Shahbaz and Sinha 2019). Caution is needed to deviate from fossil fuel–based energy-driven economy to renewables, which are cleaner and friendly to the environment and ecosystem at large. Interestingly, given that Turkey economy is on stride to meet her energy and environmental target as well as being a signatory to most environmental treaties like the Kyoto protocol makes it energy intensification less impactful on the environment even though is negligible as we observe an inverse relationship between energy consumption and EFP over the sampled period. This is the right step in the right direction and worthy of commendation. Although more giant strides are needed given the current level of pollutant emissions and huge energy deficit in the country. The fitted model is stable and free from flaws of classical linear regression (CLRM) assumptions. Figure 4 reports the stability test and shows that the model is properly fitted and stable for policy framework direction.

**Table 9:** ARDL bounds test result

**Test statistic**	**Value**	**k**
*F* statistic	6.92*	3
**Critical value bounds**		
**Significance**	**I(0) Bound**	**I(1) Bound**
10%	3.47	4.45
5%	4.01	5.07
2.50%	4.52	5.62
1%	5.17	6.36

Asterisk (*) denotes 1% significance level of rejection. Author’s compilation

The fact that regression does not translate into causality analysis is the need for causality analysis in Table 10. The TY causality test is necessary to give the predictability ability of a variable’s contemporaneous value and its past realization on another variable over a defined period. Table 10 shows that a two-way causality exists between a wave of globalization and environmental degradation (EFP), same for EFP and GDP. Similar feedback causality flow runs from economic expansion to energy consumption and economic expansion drive globalization All causality results have diverse implications. For instance, the energy consumption–induced pollutant emission through (EFP) is instructive given that the economy is emerging and needs the energy to drive manufacturing industry service industry and other sectors. Thus, there is a need for a gradual paradigm shift from nonrenewable energy sources to renewables like hydro and photovoltaic, which is encouraged (Bekun et al.,2019b; Emir and Bekun 2019). We also see globalization driving economic growth, energy intensification, and environmental degradation. This suggests that officials of the Turkish economy will manage the economy with caution such as to avoid the adverse effect of all dimensions of globalization be it social, economic, and political (Table 11).

**Table 10:** ARDL result for long and short-run

LNEFP = f (LNEU, LNGLO, LNRGDP)				
Variable	Coefficient	Std. error	t-statistics	Probability
Short-run result				
ECT (− 1)*	− 0.5829*	0.1021	− 5.7068	0.0000
∆LNEU	− 0.3746	0.2916	− 1.2844	0.2064
∆LNGLO	− 0.6540	0.4986	− 1.3115	0.1972
∆LNRGDP	0.3500	0.2631	1.3300	0.1910
Long run result				
LNEU	− 0.4275***	0.2504	− 1.7076	0.0951
LNGLO	0.6405***	0.3215	1.9921	0.0529
LNRGDP	0.7190*	0.0269	26.6698	0.0000

Author’s compilation. Asterisks (*, **, ***) denote 1%, 5%, and 10% significant level of rejection, respectively

**Table 11:** Toda-Yamamoto causality analysis

Excluded	Chi-sq	df	Prob.
Dependent variable: EFP			
EU	7.259774	2	0.0265**
GLO	13.93814	2	0.0009*
RGDP	12.54445	2	0.0019*
All	30.33783	6	0.0000*
Dependent variable: EU			
EFP	23.75400	2	0.0000*
GLO	2.415327	2	0.2989
RGDP	18.89115	2	0.0001*
All	32.09976	6	0.0000*
Dependent variable: GLO			
EFP	25.31087	2	0.0000*
EU	10.79425	2	0.0045*
RGDP	0.272536	2	0.8726
All	27.15200	6	0.0001*
Dependent variable: RGDP			
EFP	9.494295	2	0.0087*
EU	2.309845	2	0.3151
GLO	0.271829	2	0.8729
All	31.20426	6	0.0000*

Author’s compilation. Asterisks (*, **, ***) stand for 1%, 5%, and 10% significance level of rejection, accordingly

## Conclusion

The interconnectedness of the world comes with its implications on energy consumption and the environment. This has made all economies around the globe connected in terms of integration of financial systems, trade volumes and politics, and other areas. It is on the above premise that this country-specific study for the case of Turkey investigates the implication of the interconnectedness of the world economies fondly called globalization and its direct and indirect impact on energy consumption and by extension on the environment. Analysis for the mentioned studies was retrieved from the World Bank Development Indicators for the macroeconomic variables, while for globalization variables were obtained from the KOF Swiss Economic Institute Database over an annual period of 1970–2017. The operational model was constructed in a multivariate setting to avoid the omitted variable trap.

The core empirical outcomes are the validation of equilibrium relationship among the outlined variables over the investigated period a reported by the recent novel combined Bayer and Hanck cointegration methodology, which was also supported by the Pesaran’s ARDL bounds test. Further empirical revelations show that energy intensification in the face of an aggressive wave of globalization induces a reduction in environmental quality in Turkey. This is revealing and indicative to Turkish government officials that formulate and design environmental regulations in line with macroeconomic indicators like the real economic output. This outcome necessitated the following policy directions. First, the need to adequately manage the current pace of economic growth without compromising the quality of the environment. This can be achieved via the embracing and paradigm switch from fossil fuel energy sources in the Turkish energy portfolio that pollute the environment to new energy technologies like renewables that are reputed to be cleaner and more environmentally friendly. This is one sure way to guarantee a disentangle of economic growth from pollutant emissions. Second, there is an urgent need for a pragmatic step on the government to reinforce her commitment to environmental commitment like the Kyoto protocol and other indigenous energy goals/targets. This is the pathway to engender achievement of the sustainable development goals targets 2030 of cleaner energy for all. Further other studies can explore the theme with other covariates not accounted for in the current study such as demographic indicators like population and democracy among others. A reinvestigation of the theme is also of value to the extant literature for other EU candidate member to follow the trajectory on the pollutant-income nexus. Finally, the need to account for asymmetry in the econometric buildup is lacking, which is a gray area of direction for other scholars.

## Abbreviations

ADF: Augmented Dickey-Fuller unit root test
ADRL: Autoregressive distributive lag
AIC: Akaike information criterion
BH: Bayer and Hanck cointegration methodology
CO_2_: Carbon dioxide
EFP: Ecological footprint
EKC: Environmental Kuznets curve hypothesis
FPE: Final prediction error
GDP: Gross domestic product
GHG: Greenhouse gas
GNI: Gross national income
HQ: Hannan Quinn information criterion
PP: Phillips-Perron unit root test
SIC: Schwarz information criterion
UECM: Unrestricted error correction model
ZA: Zivot and Andrews unit root test

## Footnotes

1 See Table 1 for details summary on the theme under consideration
